# Exact median-tree inference for unrooted reconciliation costs

**DOI:** 10.1186/s12862-020-01700-w

**Published:** 2020-10-28

**Authors:** Paweł Górecki, Alexey Markin, Oliver Eulenstein

**Affiliations:** 1 University of Warsaw, Faculty of Mathematics, Informatics and Mechanics, Banacha 2, Warsaw, 02-097 Poland; 2 Department of Computer Science, Iowa State University, Atanasoff Hall 212, Ames, 50011 USA

**Keywords:** Median tree, Reconciliation, Gene duplication, Gene loss, Deep coalescence, Exact solution

## Abstract

**Background:**

Solving median tree problems under tree reconciliation costs is a classic and well-studied approach for inferring species trees from collections of discordant gene trees. These problems are NP-hard, and therefore are, in practice, typically addressed by local search heuristics. So far, however, such heuristics lack any provable correctness or precision. Further, even for small phylogenetic studies, it has been demonstrated that local search heuristics may only provide sub-optimal solutions. Obviating such heuristic uncertainties are exact dynamic programming solutions that allow solving tree reconciliation problems for smaller phylogenetic studies. Despite these promises, such exact solutions are only suitable for credibly rooted input gene trees, which constitute only a tiny fraction of the readily available gene trees. Standard gene tree inference approaches provide only unrooted gene trees and accurately rooting such trees is often difficult, if not impossible.

**Results:**

Here, we describe complex dynamic programming solutions that represent the first nonnaïve exact solutions for solving the tree reconciliation problems for unrooted input gene trees. Further, we show that the asymptotic runtime of the proposed solutions does not increase when compared to the most time-efficient dynamic programming solutions for rooted input trees.

**Conclusions:**

In an experimental evaluation, we demonstrate that the described solutions for unrooted gene trees are, like the solutions for rooted input gene trees, suitable for smaller phylogenetic studies. Finally, for the first time, we study the accuracy of classic local search heuristics for unrooted tree reconciliation problems.

## Background

Phylogenetic trees visualize estimates of the evolutionary relationships among multiple biological entities such as molecular sequences, genomes, and species. For biologists, such trees present a fundamental tool for analyzing how distinct biological entities have evolved but are full of complexities and seemingly irreconcilable differences [1]. The implications and potential applications of phylogenetic analyses are widespread and are concerning a wide variety of central research areas including biology, ecology, epidemiology, and conservation biology (e.g., [2–6]).

Conventional phylogenetic tree inference samples an individual gene (i.e., a gene family) for a collection of species and reconstructs the evolutionary history, called a *gene tree*, of this gene. The gene is a portion of the species’ genomes, and it is assumed that the corresponding gene tree is mimicking the evolution of the species. Accordingly, the gene tree is identified with the species tree.

However, it is well-recognized that distinct gene families frequently yield incongruent gene trees due to intricate evolutionary processes which are affecting genomic locations of the genes in different ways [7, 8]. Such processes include gene duplications, gene loss, and deep coalescence. Consequently, identifying a gene tree with its species tree can result in largely misleading phylogenetic analyses [9, 10].

Today, algorithmic advances have given rise to powerful computational tools for inferring credible species tree estimates from collections of discordant gene trees by solving median tree problems [11]. Given a set of gene trees, *median tree problems* (also referred to as supertree problems [12]) seek a tree, called a *median tree*, that is minimizing the overall cost function in regards to the input trees (using a problem-specific cost function). Such cost functions, for example, include the classic and well-studied tree reconciliation costs, which are informed by evolutionary models that explain incongruence between gene trees and species trees. Tree reconciliation costs account for the minimum number of evolutionary events required to reconcile the discordance between a gene tree and a species tree, where both trees are rooted. For the *tree reconciliation costs*, we consider here the fundamental evolutionary events of gene duplication, gene loss [13–16], and deep coalescence [17]. The median tree problems under the tree reconciliation costs, which we refer to as *(median) tree reconciliation problems*, will be the focus of this work.

Similar to the in practice established median tree problems [11], the tree reconciliation problems are also NP-hard [18, 19], and therefore, are frequently addressed by local search heuristics [20–25]. The challenging task of such heuristic approaches is to efficiently search a globally optimal species tree in a solution landscape that is literally of astronomical size (i.e., the search space increases super-exponentially with the number of taxa [26]). Moreover, this landscape has typically numerous local optima that can easily trap heuristic approaches [27]. So far, these local search heuristics lack any provable correctness or precision [28, 29].

To obviate such heuristic uncertainties, exact dynamic programming solutions have been described for the tree reconciliation problems [20, 28]. These solutions are asymptotically significantly faster than the naïve enumeration of the solution space allowing to compute median trees suitable for smaller studies. Furthermore, such exact solutions have demonstrated that local search heuristics can fail to find optimal solutions for even small-sized studies (i.e., eight species [30]). This makes exact dynamic programming solutions the most appealing choice for phylogenetic studies involving smaller numbers of taxa (e.g., up to 25 taxa [31]).

Unfortunately, the exact dynamic programming solutions are not applicable to the large number of unrooted gene trees that are typically inferred from molecular sequences. Standard tree inference methods that are used in practice, such as maximum parsimony or maximum likelihood, infer only unrooted gene trees, and identifying credible rootings in such trees is often challenging, if not unattainable [32, 33]. Outgroup rooting, for instance, can produce inaccurate rootings when evolutionary events cause heterogeneity in the gene trees. Rooting gene trees under the molecular clock hypothesis, or likewise by using midpoint rooting, also can result in an error when there is a molecular rate variation throughout the tree [34, 35].

However, there has been an increased interest in rooting unrooted gene trees using a credibly rooted species tree such that a chosen tree reconciliation cost is minimized [36–38]. The corresponding extension of the standard reconciliation costs to costs between an unrooted gene tree and a rooted species tree are well-studied and referred to as the *unrooted gene tree reconciliation cost*. For example, consider the standard deep coalescence cost defined between a rooted gene tree and a rooted species tree; the *unrooted deep coalescence cost* between an unrooted gene tree and a rooted species tree is then defined as the minimum rooted deep coalescence cost between any rooting of the unrooted gene tree and the species tree (i.e., the minimum cost over all rootings).

Naïvely, the tree reconciliation problems for the unrooted gene tree reconciliation costs can be solved by a complete enumeration of the input trees’ rooting scenarios and selecting a scenario that results in a median tree with the minimum tree reconciliation cost using an exact dynamic programming solution for rooted input gene trees. However, the super-exponential runtime increase caused by the enumeration makes this approach unsuitable even for smaller sized phylogenetic studies involving, e.g., more than five gene trees (see the “Scalability analysis” section).

Here, we describe an exact and non-naïve method, termed UrExact, for solving the tree reconciliation problems for complete^1^ and unrooted input gene trees, i.e., using the unrooted reconciliation costs. Perhaps surprisingly, UrExact has the same asymptotic runtime as the best know exact method [28], termed RExact, for the tree reconciliation problems for rooted input gene trees. In practice, however, gene trees also can be incomplete. This can be addressed by filling in the gene trees (e.g., [29]), and then applying UrExact. Another option is to apply the *minus method* [39]; that is when computing the pairwise unrooted reconciliation costs between a gene tree and a candidate species tree, the species tree is constrained to the host-species of the gene tree’s leaf-genes. We show how to handle the minus method in the “Results” section, which requires an extension of our approach. Further, using comparative experimental studies, we showcase the runtime differences between the naïve enumeration solution using RExact and our solutions in practice. These studies demonstrate that, in contrast to the naïve solution, UrExact is suitable for exact inference on smaller phylogenetic studies (i.e., up to 11 taxa for 100 input gene trees). Finally, we study the accuracy of classic local search heuristics for unrooted median tree reconciliation problems, and demonstrate that it typically requires a large number of independent heuristic runs to find an exact median tree for already small scale phylogenetic studies. This suggests that such heuristic approaches may often not be suitable for credible phylogenetic studies, in particular for large-scale studies.

### Related work

Our exact dynamic programming solution for the unrooted tree reconciliation problems, UrExact, are building on research areas related to (i) the tree reconciliation costs, (ii) the time complexities of the tree reconciliation problems, and (iii) the unrooted tree reconciliation costs. Therefore, here we will provide more detail for each of these areas.

#### Tree reconciliation costs

The pioneering work of Goodman et al. [15], almost 40 years ago, introduced tree reconciliation as a fundamental approach of explaining discord between a gene tree and a species tree by evolutionary means. *Tree reconciliation* is an approach that is embedding a rooted gene tree into a rooted species tree using a *mapping function* that relates every gene in the gene tree to the most recent species in the species tree, referred to as the *gene’s host-species*, that could have contained the gene. In practice, the mapping of a gene tree’s leaf-genes is known to be the species from which the genes were sampled. The mapping function allows identifying the evolutionary events gene duplication, gene loss, and deep coalescence. *Gene duplications* are genes in the gene tree that have the same host-species as one of their children. *Gene losses* are accounted for by the maximum subtrees in the species tree that have no host-species. Associated with the number of these events are the tree reconciliation costs which are termed *gene duplication cost*, *gene loss cost*, and *gene duplication and loss cost* accounting for the number of gene duplications, gene losses, and gene duplications plus losses, respectively. Another classic reconciliation cost is the *deep coalescence cost* that accounts for each edge in the species tree the number of embedded lineages from the gene tree minus one, which are assumed to be the additional lineages caused by deep coalescence events [17].

#### Time complexity of the tree reconciliation problems

For rooted input gene trees, the tree reconciliation problems are NP-hard under the costs for (i) gene duplication, gene loss, and gene plus loss [18], and (ii) deep coalescence [19]. However, RExact can solve these problems in *O*(3^*n*^*mn*/*b*) time and with *O*(2^*n*^+*mn*/*b*) space, using dynamic programming, a bit-vector encoding of size *b* (ideally representing the register size of the computation machine used) and Gray encoding, where *n* is the number of input trees and *m* the number of unique rooted splits in the input trees [28]. The time complexities of the tree reconciliation problems for unrooted input gene trees are open, though, we conjecture that the problems are NP-hard.

#### Unrooted tree reconciliation costs

Initially, all gene tree parsimony costs were defined only for comparing rooted gene trees with rooted species trees. [15, 17]. However, in general, unrooted trees can be compared with rooted trees by identifying the rootings of the unrooted tree that is minimizing any provided cost function between a pair of rooted trees. Further, the gene tree parsimony costs satisfy the plateau property, which is sufficient for the linear time identification of all optimal rootings and rooting costs in the unrooted gene tree. The *plateau property* is satisfied when all optimal rootings of the unrooted gene tree form a subtree in this tree, and the rootings along every path toward a leaf have monotonically increased costs [38].

## Methods

### Basic definitions

A *rooted tree* is a rooted binary tree whose leaves are labeled by species names (not necessarily uniquely). Let *T* be a gene tree. By *L*(*T*) we denote the set of all leaf labels (i.e., species) present in *T*. The root of *T* is denoted root(*T*). A node *n* is called *internal* if it has two children, which are denoted by Ch(*n*). A cluster of a node *g*, denoted by clu(*g*), is the set of leaf labels *belowg*. A *species tree* is a rooted tree whose leaves are *uniquely* labeled (that is, there are no two leaves with the same species label).

Let *S*=〈*V*_*S*_,*E*_*S*_〉 be a species tree. For nodes *a*,*b*∈*V*_*S*_, by *a*+*b* we denote the least common ancestor of *a* and *b* in *S*. We also use the binary order relation *a*≤*b* if *b* is a node on the path between *a* and root(*S*) (note that *a*≤*a*). Two nodes *a* and *b* are called *siblings* if they are children of *a*+*b*.

For a rooted tree *G*, called here a *rooted gene tree*, and a species tree *S* such that *L*(*G*)⊆*L*(*S*), a *least common ancestor mapping*, or lca-mapping, is a function from the nodes of *G* to the nodes of *S* such that *M*(*g*)=*s* if *g* and *s* are leaves with the same label, or *M*(*g*)=*M*(*a*)+*M*(*b*) if *g* is an internal node of *G* such that *a* and *b* are the children of *g*.

An *unrooted gene tree* is a tree whose internal nodes are of degree 3 and species names label the leaves. An unrooted gene tree *G* can be rooted by placing the root on an edge *e* (that is, by subdividing edge *e* with a new node *ρ* and designating it to be the root). Such a *rooting* (i.e., a resulting rooted gene tree) is denoted *G*_*e*_.

### Cost functions

Now, we introduce several cost functions used when reconciling a rooted gene tree *G* and a species tree *S*. An internal node *g*∈*V*_*G*_ is a *(gene) duplication* if *M*(*g*)=*M*(*g*^′^) for *g*^′^ a child of *g*. The total number of gene duplications is called *duplication cost* and denoted by D(*G*,*S*). The *deep coalescence* cost is defined as follows [17]: $\text {DC}(G,S)=\sum _{a,b\textrm {\ siblings in\ }G}(|\pi (M(a),M(b))| - 1)$, where *π*(*x*,*y*) is the set of all nodes on the shortest path connecting *x* and *y* in *S*. Note that the standard definition of the *deep coalescence* cost function [40] simply differs by 1−|*V*_*G*_| from our definition, which is a constant value for a fixed *G*, and the results presented in this work can be easily adapted for the standard definition. Next, we define the *the loss cost* based on the formula derived in [19]: L(*G*,*S*)=DC(*G*,*S*)+2·D(*G*,*S*)−|*V*_*G*_|+1 and *the duplication-loss cost*: DL(*G*,*S*)=D(*G*,*S*)+L(*G*,*S*).

The above cost functions can be naturally extended to unrooted gene trees. For every cost function *c* defined above for rooted gene trees, we define its unrooted counterpart as min*e*∈*E*(*G*)*c*(*G*_*e*_,*S*). For convenience, we adopt the same notation (i.e., D,DC,L,DL) to denote unrooted cost functions. The edge *e*, such that *G*_*e*_ has the minimal cost *c*(*G*_*e*_,*S*), is called *optimal* (for *c*).

For a given rooted or unrooted gene tree *G* and a species tree *S*, *c*(*G*,*S*) can be computed in linear time [41, 42].

### Problems

We say that a species tree *S* is *over a set of speciesI* if *L*(*S*)=*I*. Let *Q* be a collection of unrooted gene trees $G_{1},G_{2},\dots,G_{n}$. By *L*(*Q*) we denote the set of all species present in the trees of *Q*. We extend the notion of the cost function to collections of gene trees. For a given species tree *S* over *L*(*Q*), by *c*(*Q*,*S*) we denote the total cost $\sum _{G\in Q}c(G,S)$.

#### **Problem 1**

(uMinST - Minimal Species Tree) Given a collection of unrooted gene trees *Q* and a cost function *c* find a species tree *S*^min^ that minimizes the total cost *c*(*Q*,*S*) in the set of all species trees *S* over *L*(*Q*).

We call a species tree *S*^min^*minimal* (for *Q* and *c*) and we denote the respective minimal cost by *c*^min^(*Q*). We also define a simpler variant of the previous problem that does not require finding a tree *S*^min^ explicitly.

#### **Problem 2**

(uMinCC - Minimal Cost Computation) Given a collection of unrooted gene trees *Q* and a cost function *c* compute *c*^min^(*Q*).

Similarly, we define problems for the collections of rooted trees, called rMinST and rMinCC, respectively. Note that [28] provides a dynamic programming solution to rMinST and rMinCC.

### Solution to rMinST and rMinCC - overview

In this section, we show how rMinCC is solved in [28]. Any internal node *v* of a gene or species tree determines a split *A*|*B*, where *A* and *B* are the clusters of children of *v*. For a collection *Q* of rooted gene trees let *r*(*Q*) be the multiset of all rooted splits present in trees of *Q*. We also set *r*(*T*) to be *r*({*T*}) for any rooted tree *T*. For brevity, here we present the dynamic programming formulations only for D and DC costs. For DL and L, please refer to [28].

For a collection of gene trees *Q* and a species *s*, *Λ*(*Q*,*s*) is the total cost contribution of the nodes from *Q* to a leaf of some species tree over *L*(*Q*) labeled by *s*. Let now *X* and *Y* be two disjoint sets of species. Then *Γ*(*Q*,*X*,*Y*) is the total cost contribution of the nodes from *Q* to an internal node *v* of some species tree over *L*(*G*) such that the cluster of *v* is *X*∪*Y* and *v* has two children whose clusters are *X* and *Y*. Given a species leaf *s* and a split *X*|*Y* and a cost *c*∈{D,DC,DC}:

$$\begin{array}{@{}rcl@{}} \Lambda^{c}(Q,s)& =& \sum_{q\in r(Q)}\lambda^{c}(q,s), \\ \Gamma^{c}(Q,X|Y)& =& \sum_{q\in r(Q)}\gamma^{c}(q,X|Y), \end{array} $$

where

$$\begin{aligned} \lambda^{\mathrm{D}}(A|B,s)&=\lambda^{\text{DL}}(A|B,s)=\mathbf{1}\left[A\cup B=\{s\}\right], \\ \lambda^{\text{DC}}(A|B,s)&=\mathbf{1}\left[\exists i \colon A_{i}=\{s\}\neq A_{i+1}\right] \\ \gamma^{\mathrm{D}}(A_{1}|A_{2},X_{1}|X_{2}) & = \mathbf{1}\left[A_{1}\cup A_{2}\subseteq X_{1}\cup X_{2}\wedge \exists i\colon X_{1} \nsupseteq A_{i}\nsubseteq X_{2} \right],\\ \gamma^{\text{DC}}\left(A_{1}|A_{2},X_{1}|X_{2}\right) & = \mathbf{1}\left[\exists i,j\colon X_{j} \nsupseteq A_{i}\subseteq X_{1}\cup X_{2} \nsupseteq A_{i+1} \right]. \end{aligned} $$

The above functions can be used to compute the cost as follows:

1 $$ c(Q,S)= \sum_{\substack{s \in L(S)}} \Lambda^{c}(Q,s)+\sum_{q \in r(S)} \Gamma^{c}(Q,q).   $$

The dynamic programming solution to rMinCC from [28] is as follows:

$$\Delta^{c}(Q,Z)\,=\,\!\left\{\begin{array}{ll} \Lambda^{c}(Q,s) & \text{if}\ Z=\{s\},\\ \min_{X|Y \in {\text{splits}}(Z)} \Delta^{c}(Q,X)+\Delta^{c}(Q,Y) +\Gamma^{c}(Q,X,Y) & \text{otherwise}, \end{array}\right. $$

where splits(*Z*) is the set of all splits (2-partitions) of *Z* and the solution is given by *Δ*^*c*^(*Q*,*L*(*Q*)).

### Unrooted reconciliation

We now summarize the previous structural results on reconciliation of an unrooted gene tree with a species tree. These results will be used for the design of our main dynamic programming solutions to uMinST and uMinCC problems.

Without loss of generality, we assume that every unrooted/rooted gene tree has at least 3 distinct labels and it has all labels from a species tree, i.e., *L*(*G*)=*L*(*S*)≥3. Let *G* be an unrooted gene tree and *S* be a fixed species tree. The split of the root of *S* we call *top-split*.

**Fig. 1:**
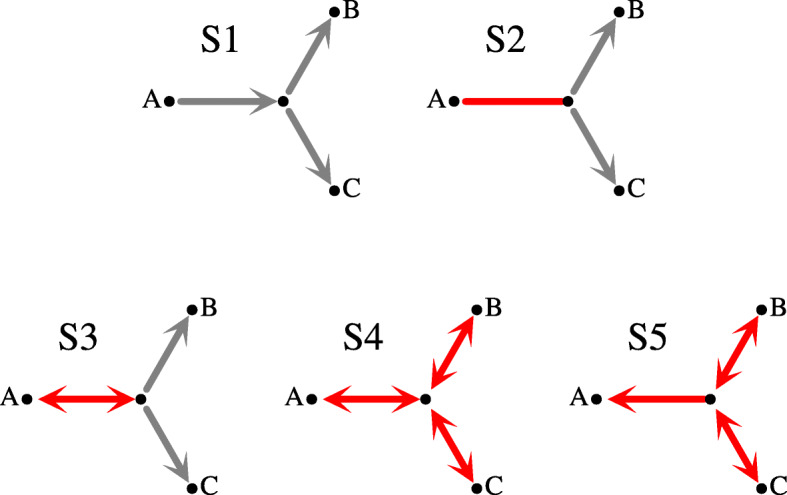
Stars S1-S5. The edges denote the relation *ζ* as follows: $A \rightarrow \bar {A}$ is $\neg \zeta (A) \wedge \zeta (\bar {A}), A \leftrightarrow \bar {A}$ is $\neg \zeta (A) \wedge \neg \zeta (\bar {A})$ and $A - \bar {A}$ is $\zeta (A) \wedge \zeta (\bar {A})$. The nodes whose clusters are *B* and *C* we call *right-hand* in a given star. A red edge is an element of the plateau, i.e., its rooting is optimal (see Thm. 1)

**Fig. 2:**
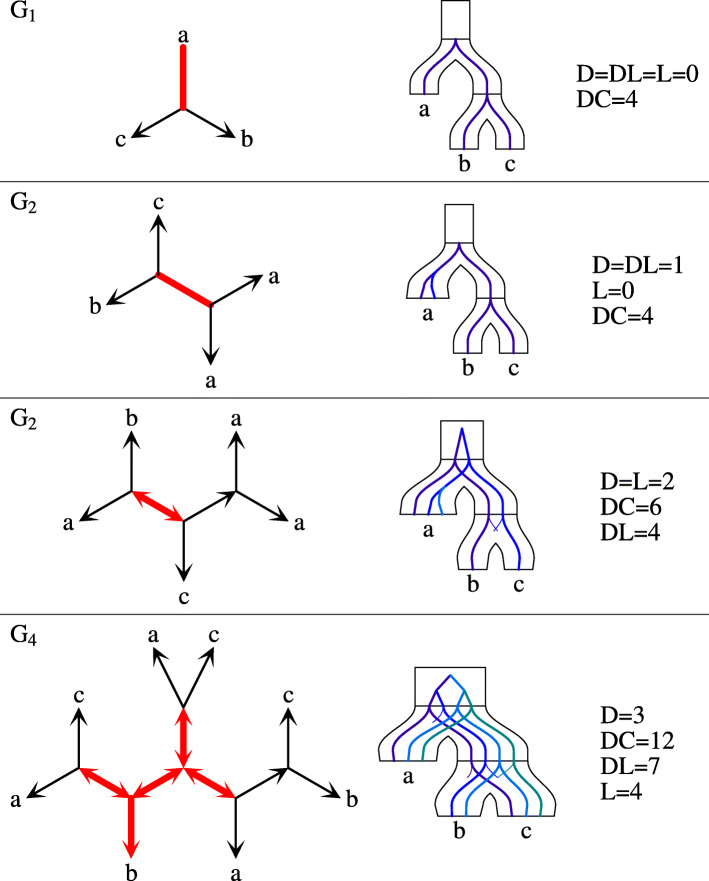
Unrooted trees and costs. Examples of four unrooted gene trees with stars reconciled with the species tree *S*=(*a*,(*b*,*c*)) having the top-split *a*|*bc*. Plateau edges are thick and red. The embeddings into *S* are obtained for one of the optimal edges located in the plateau
- if $\zeta (A) \wedge \zeta (\bar {A})$,
- if $\neg \zeta (A) \wedge \neg \zeta (\bar {A}) \wedge \zeta (B) \wedge \zeta (C)$,
- if ¬*ζ*(*A*)∧¬*ζ*(*B*)∧¬*ζ*(*C*),
- if *ζ*(*A*)∧¬*ζ*(*B*)∧¬*ζ*(*C*).

A set of species *Z* is *simple* if *Z* is a subset of a cluster from the top-split. Let *ζ*(*Z*) be a predicate that is true if *Z* is simple. Further, let *G* be an unrooted tree. Any internal node *g* of *G* determines a star *A*|*B*|*C*, where *A*, *B* and *C* are the leaves of three subtrees obtained from *G* by removing *g*. Note that *A*∪*B*∪*C*=*L*(*S*). Let $\bar {A}=B \cup C, \bar {B}=C \cup A$ and $\bar {C}=A \cup B$. Then, it follows from [42, 43], that given a top-split we have five disjoint types of stars (see Fig. 1) in unrooted gene trees (reordering of *A*, *B* and *C* may be required; e.g., the two stars in *G*_2_ from Fig. 2 are represented as *a*|*b*|*c* (or *a*|*c*|*b*) and *bc*|*a*|*a* in the context of the top-split *a*|*bc*):

- if $\neg \zeta (A) \wedge \zeta (\bar {A})$,

Given a species tree *S*, an edge of an unrooted tree *G* is *symmetric* if removing the edge from *G* creates two trees whose top-clusters are either both simple or both are not simple. The remaining edges are called *asymmetric*. E.g., all edges of S4 are symmetric, while all edges of S1 are asymmetric. We have the main theorem for unrooted reconciliation theory.

#### **Theorem 1**

(Adopted from [42–45]) If *e* is a symmetric edge in any star or *e* is the asymmetric edge from S5 then *c*(*G*_*e*_,*S*)=*c*(*G*,*S*), i.e., *e* is optimal for every cost *c*∈{D,DC,L,DL}.

The edges satisfying the conditions from the above theorem induce a connected unrooted subtree in *G* called a *plateau*. The plateau in our article equals the DL-plateau from [42], where the *c*-plateau is the graph induced by the set of optimal rooting edges for the cost *c*. Since any optimal rooting for DL is optimal for D, DC and DL [42], to solve our problems it is sufficient to focus on the plateau (or DL-plateau) rootings only. Examples are depicted in Fig. 2.

### Decomposing unrooted gene trees

Before we start with the main results, we show how given a species tree (or its top-split only), an unrooted gene tree can be decomposed into two parts: one part which is of the rooted nature and the second part related to the plateau. Note that the notion of a cost function refers to D, DC, L or DL.

We start with the following observation, which follows from Theorem 1 and stars definitions.

#### **Lemma 1**

The top-split of the species tree is sufficient to determine the optimal rooting of a given unrooted gene tree.

A *rooted subtree* of an unrooted gene tree *G* is a proper subtree of some rooting of *G*. Then, we have the following property.

#### **Lemma 2**

Let *T* be a rooted subtree of an unrooted gene tree *G* and let *S* be a species tree over *L*(*G*). *T* is a subtree in every plateau rooting of *G* if and only if the following conditions are satisfied:

- *M*(*t*)<root(*S*),
- or *M*(*t*)=root(*S*) and *t* is a not a duplication,

where *t*=root(*T*).

#### *Proof*

(<=) The statement is obvious if *t* is a leaf. We show that no edge in *T* is symmetric in *G* if *t* is an internal node in *G*. If *M*(*t*)<root(*S*) then *t* is a center of S1 or S2 and the children of *t* in *T* are right-hand in the star. Thus, the remaining edges in *T* are asymmetric (only S1 can be present in *T*) with the star-arrows directed towards the leaves of *T*. A similar property holds in the second case with the difference that *t* is a center of S3. Thus, the edges of *T* are disjoint with the plateau and *T* is a rooted subtree in every plateau rooting of *G*. (=>) If *t* is mapped below the root of *S*, the tree contains only asymmetric non-plateau edges. Otherwise, *t* is a duplication mapped to the root, then at least one edge *e*^′^ connecting *t* with its child is symmetric. Thus, *T* is not present in the rooting placed on *e*^′^. A contradiction. □

**Fig. 3:**
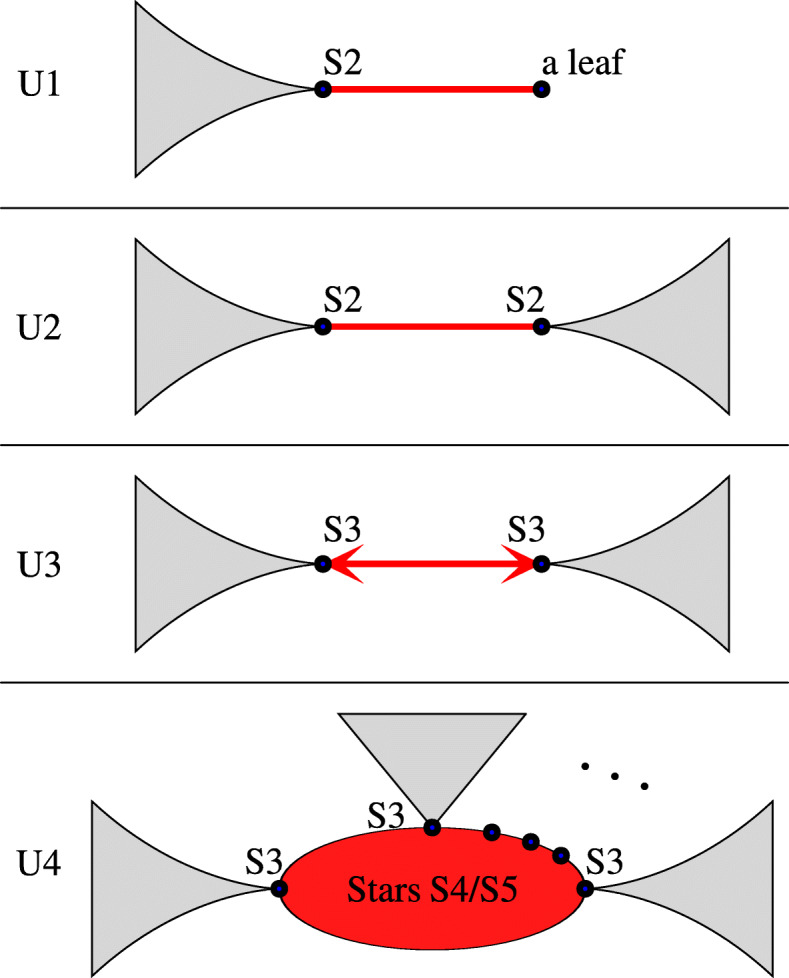
Types of unrooted trees. Four possible types of unrooted gene trees *G* (under the assumption that |*L*(*G*)|≥3 and *L*(*S*)≥3). Red color (in the center) denotes *G*^∗^ (plateau) while grey subtrees denote the rooted forest $\check {G}$. Border nodes are marked by black circles and decorated with its possible star types (when applicable). See also Fig. 2

We conclude that every unrooted gene tree *G* can be decomposed into two parts: a *plateau* *G*^∗^ and the *rooted forest*$\check {G}$ obtained from *G* by removing the internal nodes and edges of *G*^∗^. Note that *G*^∗^ is an unrooted tree with at least one edge and $\check {G}$ is a forest whose edges are asymmetric in *G*. Moreover, if $\check {G}$ contains a tree with a non-root internal node, then this node is a center of S1. The leaves of *G*^∗^, which are also the roots of $\check {G}$, are called *border* nodes. Since not every two stars can share an edge, possible topologies of stars in gene tree are limited. Figure 3 depicts all possible types of gene trees depending on the stars (see also Fig. 2).

### Cost contribution functions

For an unrooted gene tree *G* let stars(*G*) be the multiset of all stars present in *G* (similarly it is defined for collections of gene trees). Here we define the cost contribution functions for our four standard costs. For every cost, we define $\hat {\lambda }$ as the contribution of a given star to a species (i.e., a leaf of the species tree) in the context of a top-split, $\hat {\gamma }$ as the contribution of a given star to a non-root internal node of a species tree (a split *X*|*Y*) in context of a top-split, *σ* as the contribution of a given star to the root of a species tree in context of a top-split, and *ε* which is the cost correction depending on the gene tree. For an unrooted gene tree *G*, a species *s* and a top-split ⊤, $\hat {\Lambda }(G,s,\top)$ is the total cost contribution of the stars (in the context of a top-split ⊤) from *G* to a leaf of some species tree over *L*(*G*) labeled by *s*. Given two disjoint sets of species *X* and *Y* and a top-split ⊤, $\hat {\Gamma }(G,X|Y,\top)$ is the total cost contribution of the stars (in the context of a top-split ⊤) from *G* to an internal node *v* of some species tree over *L*(*G*) such that (i) the cluster of *v* is *X*∪*Y* and (ii) *v* has two children whose clusters are *X* and *Y*, respectively. Then, the total cost contributions are defined:

$$\begin{array}{@{}rcl@{}} \hat{\Lambda}^{c}(G,s,\top) &=& \sum_{a \in {\text{stars}}(G)}\hat{\lambda}^{c}(a,s,\top), \\ \hat{\Gamma}^{c}(G,X|Y,\top) & =& \sum_{a \in {\text{stars}}(G)} \left\{\begin{array}{ll} \sigma^{c}(a,\top) & X|Y=\top,\\ \hat{\gamma}^{c}(a,X|Y,\top) & \text{otherwise},\\ \end{array}\right. \end{array} $$

where the contribution functions $\hat {\lambda }^{c}, \hat {\gamma }^{c}$ and *σ*^*c*^ for *c*∈{D,DL,DC} are depicted below. Here, ⊤ is a top-split of some species tree *S*, *X*|*Y* is a split of some node from *S*, *s* is a species, *G* is an unrooted gene tree and $\mathcal {A}=A|B|C$ is a star of type *τ* from *G*.

- Duplication cost (D):
  2 $$\begin{array}{*{20}l} \hat{\lambda}^{\mathrm{D}}(\mathcal{A},s,\top)&=\mathbf{1}[B=C=\{s\} \wedge \tau \in \{\text{S1}, \text{S2}\}]  \end{array} $$
  3 $$\begin{array}{*{20}l} \hat{\gamma}^{\mathrm{D}}(\mathcal{A},X|Y,\top)&=\gamma^{\mathrm{D}}(B,C,X|Y)  \end{array} $$
  4 $$\begin{array}{*{20}l} \epsilon^{D}(G)&=1. \end{array} $$

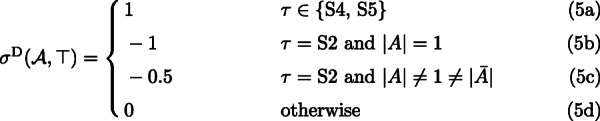
- Deep Coalescence cost (DC):
  $$\begin{aligned}{\hat{\lambda}^{\text{DC}}(\mathcal{A},s,\top)\,=\,\!\left\{\begin{array}{ll} \lambda^{\text{DC}}(B|C,s) & \text{if}\ \tau \in \{\text{S1, S3}\}\\ \lambda^{\text{DC}}(A|\bar{A},s) & \text{if}\ \tau=\text{S5}\\ \max \left\{ \lambda^{\text{DC}}(B|C,s), \lambda^{\text{DC}}(A|\bar{A},s) \right\} & \text{if}\ \tau=\text{S2} \wedge \neg (B\,=\,C \!\wedge |B|=1)\\ 0 & \text{otherwise} \end{array}\right.} \end{aligned} $$
  $$\begin{aligned}{\hat{\gamma}^{\text{DC}}(\mathcal{A},X|Y,\top)\,=\,\! \left\{\begin{array}{ll} \gamma^{\text{DC}}(B,C,X|Y) & \tau \in \{\text{S1, S3}\}\\ \gamma^{\text{DC}}(A,\bar{A},X|Y) & \tau=\text{S5}\\ \gamma^{\text{DC}}(B,C,X|Y)+ \\ \ \ + \frac{1+[|A|=1]}{2}\gamma^{\text{DC}}(A,\bar{A},X|Y) & \tau=\text{S2}\ \text{and}\ \neg (B\,=\,C \!\wedge\! |B|\!=1)\\ 0 & \text{otherwise} \end{array}\right.} \end{aligned} $$
  $$\begin{array}{*{20}l} \sigma^{\text{DC}}(\mathcal{A},\top)&=0, \\ \epsilon^{\text{DC}}(G)&=0. \end{array} $$
- Loss and Duplication-Loss cost; for *c*∈{L,DL}, let *ω*_L_=2 and *ω*_DL_=3 in
  $${}{\begin{aligned} \hat{\lambda}^{c}(\mathcal{A},s,\top)&=\hat{\lambda}^{\text{DC}}(\mathcal{A},s,\top)+\omega_{c}\cdot \hat{\lambda}^{\mathrm{D}}(\mathcal{A},s,\top) \\ \hat{\gamma}^{c}(\mathcal{A},X|Y,\top)&=\hat{\gamma}^{\text{DC}}(\mathcal{A},X|Y,\top) +\omega_{c}\cdot \hat{\gamma}^{\mathrm{D}}(\mathcal{A},X|Y,\top) \\ \sigma^{c}(\mathcal{A},\top)&=\sigma^{\text{DC}}(\mathcal{A},\top) +\omega_{c}\cdot \sigma^{\mathrm{D}}(\mathcal{A},\top) \\ \epsilon^{c}(G)&=1-|V_{G}|+\omega_{c} \end{aligned}} $$

The next theorem defines how these functions can be used to compute the unrooted cost given an unrooted gene tree and a species tree. This result is the unrooted analogue of Eq. (1).

#### **Theorem 2**

Let *G* be an unrooted gene tree with |*L*(*G*)|≥3 and *S* be a species tree over *L*(*G*). If *c* is a cost function {D,DL,L,DC} and ⊤ is the top-split of *S*, then

$$\begin{aligned} c(G,S) & =\! \sum_{s \in L(S)} \hat{\Lambda}^{c}(G,s,\top)+ \sum_{x,y \text{\ siblings in} S} \hat{\Gamma}^{c}(G,{\text{clu}}(x)|{\text{clu}}(y),\top)+\epsilon^{c}(G). \end{aligned} $$

#### *Proof*

Let *R* denote the right-hand side of the above formula. We start with the duplication cost and gene trees of type U4.

***Cost D vs. U4:*** The plateau of *G* contains stars S4/S5. Then, the remaining stars have type S1/S3. We show that D(*G*_*e*_,*S*)=*R*, where *e* is a plateau edge. Let *g* be an internal node of *G*_*e*_, *M*(*g*)=*s* and, if *g*≠root(*G*_*e*_), then *g* is a center of star *A*|*B*|*C* of type *τ*.

(U4.a) If *s* is a leaf. Then, *g* is a duplication and *τ* is S1 with *B*=*C*={*s*}. The case is incorporated in $\hat {\lambda }^{D}$ (see (2)). Note that *τ* cannot be S3/S4/S5 under the assumption that *L*(*S*)≥3.

(U4.b) If *s* is an internal non-root node of *S* with the split *X*|*Y*. In this case, *B*∪*C*⊆*X*∪*Y* and *X*∪*Y* is a subset of the element from ⊤. Then, *τ*=S1. In such a case, *g* is a duplication if and only if *γ*^*D*^(*B*|*C*,*X*|*Y*,⊤)=1 (See (3), where *γ*^*D*^ was defined in “Solution to rMinST and rMinCC - overview” section.

(U4.c) The remaining case is when *s*=root(*S*). We have three subcases: *τ*=S3 (border of *G*^∗^), *τ*∈{S4,S5} (internal of *G*^∗^) or *g*=root(*G*_*e*_). If *τ*=S3, then, *g* is a border node and by Lemma 2, it is not a duplication. In our formula, the contribution of stars S3 is 0. If *τ*∈{S4,S5} then *g* is a duplication. This case is contributed in (5a). Finally, if *g*=root(*G*_*e*_) then it is also a duplication counted in *ε*^D^(*G*). Note that, given a plateau with *n* internal nodes (or equivalently, *n* stars S4/S5), the total number of duplications at the root of *S*, equals *n*+1 (one duplication for each star S4/S5 plus one for the root of *G*). Thus, our formulas count exactly the number of duplications in such a case.

This completes the proof for gene trees of the type U4.

***Gene tree type U3:*** The proof is almost the same as in the previous case. The only difference is that the plateau consists of a single edge (*n*=0; no stars in the plateau). Thus, the third subcase in (U4.c) can be omitted.

***Gene tree type U1-U2:*** Now, the gene tree contains one or two stars S2 (sharing an edge) and the remaining stars are of type S1 (if present).

(U1-2.a) If *s* is a leaf, then *τ* is S1 or S2. Then, *g* is a duplication and the rest is similar to (U4.a).

(U1-2.b) The same as (U4.b) plus the case when *τ*=S2.

(U1-2.c) If *g*=root(*G*_*e*_), then it is not a duplication, and its contribution is 0.

Since there is no duplication mapped to root(*S*) if *G* contains S2, the above cases count every duplication present in *G*_*e*_. However, the presence of *ε*^*D*^(*G*)=1 which is needed for proper calculation in other cases, requires correction of the total contribution. The correction by −1 is only performed when S2 is present in *G*, but the difficulty is that *G* can have one or two such stars. This correction is embedded in (5b) and (5c) as follows. Assume that the first star S2 is *A*|*B*|*C*. If the second star S2 is present we assume it is $\bar {A}|B'|C'$, where *B*^′^∪*C*^′^=*A*.

- |*A*|=1 and *G* has only one star S2. Then, the star contributes −1 in (5b).
- |*A*|=1 and *G* has two stars S2. The first star contributes −1 from (5b), while the second star contributes 0 from (5d).
- $|A| \neq 1 \neq |\bar {A}|$. In such a case *G* has two stars S2, each contributes −0.5 from (5c).

Thus, the total correction is −1 in every case. This completes the proof for the case of duplication cost.

***Cost DC:*** The technical proof for DC is analogous to D.

***Cost DL and L***: Note that the formulas for DL and L are linear combinations of D and DC with the scalar addition of 1−|*V*_*G*_| (see “Cost functions” section). Further, the optimal rootings are shared across all costs; therefore, the proof for this case follows from D and DC proofs and the corresponding contribution formulas. □

### Dynamic programming solution to uMinCC

Let *Q* be a collection of unrooted gene trees, *c* be a cost function and *Z*⊆*L*(*Q*). Here we extend $\hat {\Gamma }, \hat {\Lambda }$ and *ε* to collections of unrooted gene trees, e.g., $\hat {\Gamma }(Q)=\sum _{G \in Q} \hat {\Gamma }(G)$ and so on. The dynamic programming formulas for the solution to the uMinCC problem is defined as follows (the superscript *c* is omitted):

$$\begin{array}{@{}rcl@{}} \Upsilon(Q,Z,\top) \,=\, \begin{array}{ll} \hat{\Lambda}(Q,s,\top) & \text{if}\ Z=\{s\},\\ \min_{X|Y \in {\text{splits}}(Z)} \Upsilon^{*}(Q,X|Y,\!\top) & \text{otherwise}, \end{array} \\ \Upsilon^{*}(Q,X|Y,\top)\,=\,\Upsilon(Q,X,\top)\,+\,\Upsilon(Q,Y,\top)+\hat{\Gamma}(Q,X|Y,\top). \end{array} $$

Informally, in the above recurrence, for each top-split *X*|*Y* and for each *Z* such that *Z*⊆*T* or *Z*⊆*T*^′^, *Υ*(*Q*,*Z*,⊤) is the minimal cost contribution of a non-plateau parts of input gene trees in the set of all species trees over *L*(*Q*) having a node *v* whose cluster is *Z*, where the contribution is only calculated for the nodes strictly descendant from *v*. The formula for *Υ*^∗^(*Q*,*X*|*Y*,⊤) extends the *Υ* by including the contribution of the nodes whose split equals *X*|*Y*. Note that the contribution of the plateau is incorporated in *Υ*^∗^ in the special case when *X*|*Y*=*Q* (see def. of $\hat {\gamma }$).

#### **Theorem 3**

Let *c* be a cost function and *Q* be a collection of unrooted gene trees such that |*L*(*Q*)|≥3, and *L*(*Q*)=*L*(*G*) for every gene tree *G* from *Q*. Then, the solution to uMinCC is min⊤∈splits(*L*(*Q*))*Υ*^∗^(*Q*,⊤,⊤)+*ε*(*Q*).

#### *Proof*

Without loss of generality, we may assume that *Q* consists of one gene tree *G*. Given a species tree *S* with top-split ⊤ and having a node *s* with a cluster *Z*, by partial *Z*-contribution of *S* we denote the partial cost defined recursively as follows:

$$\begin{aligned} c_{Z}(G,S)= \left\{ \begin{array}{ll} \hat{\Lambda}^{c}(G,s,\top) & \text{if } Z=\{s\}, \\ c_{X}(G,S)+c_{Y}(G,S)+\hat{\Gamma}^{c}(G,X|Y),\top) & \text{if }X|Y \text{ is the split of \textit{s}}. \end{array}\right. \end{aligned} $$

It follows from Theorem 2, that *c*(*G*,*S*)=*c*_⊤_(*G*,*S*)+*ε*^*c*^(*G*).

Let ⊤=*T*|*T*^′^. It is sufficient to prove that, for each *Z*⊆*T* or *Z*⊆*T*^′^, *Υ*^*c*^(*Q*,*Z*,⊤) is the minimal partial *Z*-contribution in the set of all species trees over *L*(*Q*) having a node whose cluster is *Z*. Then, it follows that *Υ*^∗^(*Q*,⊤,⊤) is the minimal partial ⊤-contribution in the set of all species trees over *L*(*Q*) having a top-split ⊤.

The proof follows by induction from Theorem. 2. We omit technical details. □

Solving uMinST follows directly from the values of *Υ*: it is sufficient to track which partitions of the cluster *Z* into *X* and *Y* under a given top-split yield the minimal value. Such partitions determine clusters in optimal species trees and they can be used to infer one or all optimal species trees.

#### **Theorem 4**

(Complexity) Given a collection of *Q* unrooted gene trees with *m* leaves and *n* species. The time complexity of the dynamic programming formula is *O*(*nm*3^*n*^) and the space complexity is *O*(3^*n*^+*m*).

#### *Proof*

We have the following identities: |stars(*Q*)|=*m*−2|*Q*|, the number of splits of |*X*|=*k* equals $\frac {1}{2}\sum _{k=1}^{n-1} \binom {n}{k} = 2^{k-1}-1$. Then, computing stars requires *O*(*m*) time, while each value of *λ*, *γ*, etc. requires *O*(*n*) time. Thus, a single $\hat {\gamma }$ or $\hat {\lambda }$ computation needs *O*(*mn*) time. Note that *Z* must be a nonempty subset of an element from ⊤ in *Υ*. Thus, the size of *Υ* array is $\sum _{k=1}^{n-1} \binom {n}{k}(2^{k}-1) \approx 3^{n}$. Similarly, the size of $\hat {\gamma }$ array is $\sum _{k=1}^{n-1}\binom {n}{k}(2^{k-1}-1) \approx 3^{n}$. Thus, the time complexity of the algorithm is *O*(*mn*3^*n*^) and the space complexity is *O*(3^*n*^+*m*). □

Now, we compare the above complexity with the naïve approach i.e., try all rooting variants to compute the minimum cost. We may assume that every gene tree has the size of *n*. Then, we have 2*n*−3 rooting variants of a single gene tree. As the REXACT requires *O*(*mn*3^*n*^) time, we need *O*((2*n*−3)^*m*^*mn*3^*n*^) time for the naïve algorithm.

### The general case when *L*(*G*)⊆*L*(*S*)

When *L*(*G*)⊆*L*(*S*), the split needed to determine the star type can be located below the root of *S*. For example, the star *ab*|*c*|*d* cannot be determined for the split *abcde*|*f*, since the set of labels of the star is a subset of an element of the split. To determine the type of a star *A*|*B*|*C* the species tree split *X*|*Y* has to satisfy the following condition: *L*⊆*X*∪*Y*, *L*∩*X*≠*∅* and *L*∩*Y*≠*∅*, where *L*=*A*∪*B*∪*C*. The split *X*|*Y* satisfying above condition will be called a *rooting* split for *L*. Then, Theorem 2 can be reformulated as follows

#### **Theorem 5**

Let *G* be an unrooted gene tree with |*L*(*G*)|≥3 and *S* be a species tree such that *L*(*G*)⊆*L*(*S*). If *c* is a cost function {D,DL,L,DC} and *T* is a split in *S* such that *T* is a rooting split for *L*(*G*), then

$$\begin{array}{@{}rcl@{}} c(G,S)&= &\sum_{s \in L(S)} \hat{\Lambda}^{c}(G,s,T)\\&+& \sum_{x,y \text{\ siblings in\ } S} \hat{\Gamma}^{c}(G,{\text{clu}}(x)|{\text{clu}}(y),T)+ \epsilon^{c}(G). \end{array} $$

#### *Proof*

Note that the rooting split is uniquely determined. The rest follows from the proof of Theorem 2. □

It follows from the above theorem that the rooting split is crucial for proper computation of the cost. Therefore, the dynamic programming formula has to be modified to capture possible different rooting splits. Let *τ* be the set of pairs 〈*a*,*R*〉, where *a* is a star and *R* is its rooting split. Then, we have the following dynamic programming formula:

$${}\Upsilon(Q,Z,\tau)= \begin{array}{ll} \sum_{\left\langle{a,R}\right\rangle \in \tau} \lambda^{c}(a,s,R) & \text{if}\ Z=\{s\},\\ \min_{X|Y \in {\text{splits}}(Z)} \Upsilon^{*}(Q,X|Y,\tau') & \text{otherwise}, \end{array}  $$

$$\begin{array}{@{}rcl@{}} \Upsilon^{*}(Q,X|Y,\tau)&=\Upsilon(Q,X,\tau)+\Upsilon(Q,Y,\tau)\\&+\sum_{\left\langle{q,X|Y}\right\rangle \in \tau} \sigma(a,X|Y)+\\ &+\sum_{\left\langle{q,R}\right\rangle \in \tau, R \neq X|Y} \gamma(a,X|Y,R), \end{array} $$

where *τ*^′^=*τ*∪{〈*a*,*X*|*Y*〉:if *X*|*Y* is a rooting split for *a* ∈ stars(*Q*) }. Now, the time complexity of the algorithm is *O*(*mn*3^2*n*^) as the *τ* is an additional component with *O*(*m*3^*n*^) possible values.

Similarly, we can adopt the dynamic programming algorithm to solve the species tree inference using the minus method in which the cost between a gene tree *G* and a species tree *S* such that *L*(*G*)⊆*L*(*S*) is defined as *c*(*G*,*S*|_*L*(*G*)_), were *S*|_*L*(*G*)_ is the species tree obtained from *S* by contracting the set of species in *S* to *L*(*G*). Then, the above dynamic programming algorithm solves the problem under the minus method setting; however, the contribution formulas require modification. For the rooted component, the formulas (*λ* and *γ*) are provided in [28], while for the plateau component (*σ*) they remain unchanged for all our cost functions.

## Results

### Scalability analysis

We present a scalability study that compares the runtimes of our proposed dynamic programming method with the previously best-known (naïve) method to solve the median tree reconciliation problems for unrooted input gene trees. Recall that, as presented in the introduction, the naïve method is enumerating all possible combinations of rootings of the input trees and solving the respective rooted problem for each such combination. The number of such combinations grows exponentially fast with the increase of the number of input trees (i.e., *n*^*k*^, where *n* is the number of taxa and *k* is the number of trees). We (i) showcase that our method is significantly more scalable than the naïve approach and then (ii) we study the scalability of our method under conditions, which are infeasible for the naïve method.

#### Experimental setup

Both the developed here *unrooted* exact method and the previously introduced *rooted* exact method [28] were implemented in Python. Recall that the naïve method uses the rooted exact method as a subroutine. The overall naïve method was implemented in Python as well.

The scalability studies were performed under Ubuntu 14.04.5 LTS with 1.4GHz CPUs.

#### Discussion

To compare the implementations of UREXACT and the naïve method we study the scalability on input instances with a fixed number of taxa (*n*=9) and a varying number of input trees (*k*∈{2,3,4,5}). The input trees for each such instance were generated randomly using the pure-birth process; the roots were then removed from trees. For consistency, we generated five datasets for each different *k*.

The scalability results (runtimes averaged over five datasets) are presented in Table 1. Note that for *k*=2 and *k*=3 the naïve approach proved to be somewhat faster than UREXACT (this can be explained by). However, already for *k*=5, the exponential run-time growth of the naïve approach resulted in the approximately 120-fold advantage of the UREXACT method. Further, naïve method could not complete for *k*=6 in under 24 hours.

**Table 1:** Average runtimes of the UREXACT and naïve methods; (s) stands for seconds, (m) for minutes, and (h) for hours; *k* denotes the number of trees

	*k*=2	*k*=3	*k*=4	*k*=5
UrExact	75.9(s)	113.8(s)	2.6(m)	0.05(h)
naïve	7.7(s)	111.7(s)	26.1(m)	6(h)

As shown above the naïve method is infeasible for more than five input trees; it is important to note that most of phylogenetic studies in practice involve significantly more than five input trees. Next, we present the scalability study of UREXACT under more realistic conditions. Namely, we generated several input datasets with the number of trees *k*=20 and *k*=100 and the number of taxa varying from *n*=5 to *n*=11. Similar to the previous study, to present consistent results we generated five datasets for each combination of *k* and *n* and averaged the runtime of our method over those five instances; the input trees were generated using the pure-birth process.

The average runtime results are depicted in Table 2. It shows that our method was able to complete computations of exact median trees for all generated datasets. In the worst case (*n*=11 and *k*=100) UREXACT took about 26 hours to complete. Note that Tables 1 and 2 confirm that the scalability of the presented method grows *linearly* with the number of trees (as implied by the asymptotic analysis in “Dynamic programming solution to uMinCC” section). This is a significant achievement, given that the naïve approach assumes exponential growth.

**Table 2:** Average runtimes of the UREXACT method; (s) stands for seconds, (m) for minutes, and (h) for hours; *n* denotes the number of taxa

	*n*=5	*n*=6	*n*=7	*n*=8	*n*=9	*n*=10	*n*=11
*k*=20	0.7(s)	4.1(s)	24.9(s)	154.2(s)	11.7(m)	66.8(m)	7.8(h)
*k*=100	3.1(s)	19.3(s)	116.2(s)	678.8(s)	62.1(m)	314.7(m)	25.7(h)

### Accuracy of heuristics

Local search heuristics have become a standard when addressing median tree problems (e.g., [20, 46–49]). Consequently, analyzing the accuracy of such heuristics is of critical importance. Recent algorithmic advances in exact computing solutions for median tree reconciliation problems made it possible to evaluate the accuracy of local search heuristics addressing these problems [31, 50]. However, these algorithmic advances address only tree reconciliation problems for rooted input trees. Our presented algorithms solve the tree reconciliation problems exactly for unrooted trees with up to 11 taxa (see “Scalability analysis” section). Thus, for the first time, these algorithms (implemented in UREXACT) make it possible to analyze the accuracy of local search heuristics for reconciliation problems for unrooted input trees suitable for smaller phylogenetic studies. Here, we analyze the accuracy of URHEUR [37], one of the theoretically most advanced local search heuristics for unrooted tree reconciliation problems that are implementing the elementary “valley-terraces theorems” [38].

A local search heuristic initiates its median tree search for a given set of input trees under some distance from an additionally provided starting tree. The heuristic searches the local neighborhood of the candidate tree for a tree with the minimum overall distance to the input trees, which constitutes a local search step. Then, the tree found in a local search step becomes the starting point for the following local search step, and so on, until a local minimum is reached, which is reported by the heuristic. The local neighborhood of a tree is the set of trees into which it can be transformed by applying at most one tree edit-operation of a fixed type.

URHEUR implements the above described local search heuristic for median tree reconciliation problems under the gene duplication (D) and the duplication-loss (DL) costs, using the classic Rooted Nearest Neighbor Interchange tree edit operation for rooted trees [51] to define the local neighborhood. Further, URHEUR offers the option to choose the starting tree to be randomly selected under the Yule random tree model, called *random mode*, or using a cluster-based approach *called clusters mode*. The clusters mode selects the clusters for the starting tree following the greedy consensus strategy when generalized to incomplete input trees. If the resulting tree is not binary, then the tree is binarized randomly by adding clusters under the Yule model.

#### Experimental setup

Similarly to the scalability study in “Scalability analysis” section, we generated 5 independent gene tree sets (datasets) for each *n*=5,6,…,11 (where *n* indicates the number of taxa). Each such dataset contains 20 trees generated via the pure-birth process.

Since URHEUR is a randomized local search method that performs a single local search run, we executed it 10000 times on each dataset. Additionally, we executed UREXACT on each dataset. That way we were able to evaluate the *accuracy* of URHEUR. That is, for each dataset we computed how many times (out of the 10000 runs) did the heuristic find a *global optimum* (i.e., a true median tree). We report the resulting percentage (averaged over 5 independent datasets for each *n*) as the *accuracy* of the heuristic.

#### Results and discussion

**Fig. 4:**
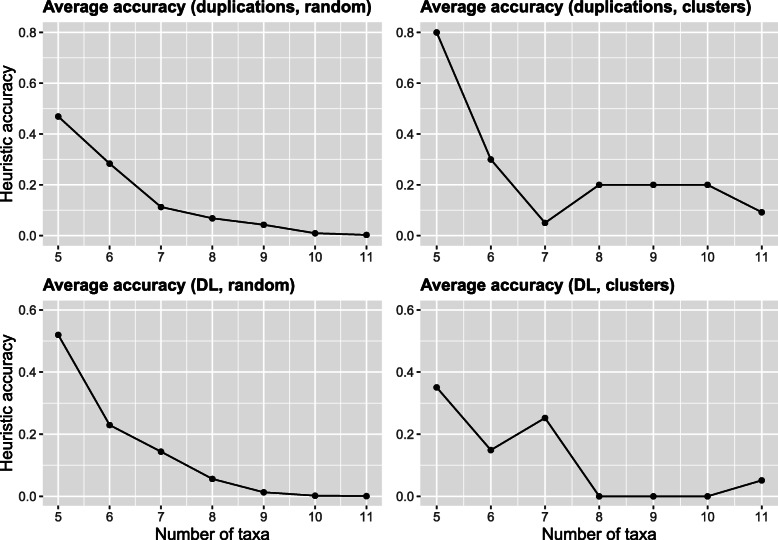
Heuristic-exact comparison. Demonstrates the accuracy of URHEUR, enabled by the exact inference via UREXACT. Each plot title in parenthesis indicates the cost function (duplications/DL) and the heuristic mode (random/clusters)

Figure 4 showcases the obtained results, which were compute using a standard laptop in less than 24 hours. Note that we performed our comparison on the examples of the classic duplication (D) and duplication-loss (DL) costs. Further, we executed the heuristic in the modes random and cluster.

Figure 4 demonstrates that, while the accuracy of the heuristic is quite high for smaller taxa numbers, it quickly deteriorates. That is, it typically requires a large number of independent runs of URHEUR to find a global optimum for *n*≥9. Further, URHEUR is never guaranteed to find a global optimum; for example, it never does so in the (DL, clusters) condition for *n*∈{8,9,10}.

The trends shown in Fig. 4 suggest that, for larger number of taxa, it is crucial to have exact solutions, since the heuristic is never guaranteed to find a global optimum.

## Discussion

In this work, we presented the first method that is capable of computing exact median trees (under tree reconciliation costs) from collections of unrooted gene trees. In particular, we introduced a novel algorithm that solves median tree reconciliation problems under the gene duplication, deep coalescence, gene loss, and gene duplication-loss cost functions. This dynamic programming solution represents the first step towards enabling the efficient inference of large-scale median trees using the cluster-constrained paradigm (for more details, see ASTRAL [52]).

Our method, UREXACT, represents a complex dynamic programming approach; it builds on top of the previously introduced REXACT method [28] via employing, and extending, the plateau properties (see “Unrooted tree reconciliation costs” section). The main observation used for the design of the algorithm is that an optimum rooting of an input gene tree only depends on the top two clusters of the species tree (i.e., the clusters/clades of the two nodes immediately below the root); we refer to such top two clusters as the *top-split*. Then the dynamic programming formulas were expressed through a comprehensive analysis of different configurations of plateaus (and the corresponding *star types* [38]) that could appear in the input gene trees. For each unrooted reconciliation cost function, we propose a novel way to compute its cost using contribution functions that depend only the star and the split from the species tree. Having this, we design a unified dynamic programming algorithm, that can be parametrized by the set of contribution functions. Given the intricacy of the contribution functions’ formulations, we provide a formal proof of their correctness focusing mainly on the gene duplication cost function, allowing their verification.

It is important to note that our method UREXACT runs in *O*(*mn*3^*n*^) time for complete input gene trees, which is asymptotically the same as for REXACT. We show how to handle the minus method in “The general case when *L*(*G*)⊆*L*(*S*)” section, adding a factor of *O*(*m*3^*n*^) to the overall time complexity.

Further, we showcase two scalability studies demonstrating the ability of UREXACT. In the first study, we compare UREXACT with the naïve method (implemented via enumerating all possible rootings of the input trees) and demonstrate that the naïve approach becomes infeasible when the number of input trees exceeds five (which is a very small number of trees for studies in practice). The second study demonstrates the scalability of UREXACT under more realistic conditions – we fix the number of input trees to be either 20 or 100. UREXACT was able to compute exact median trees with up to 11 taxa.

Finally, we evaluated the significance of UREXACT in comparison to an inexact, but fast, local search heuristic, URHEUR [37]. In particular, having the exact median tees computed by UREXACT, we were able to evaluate the accuracy of URHEUR on up to 11 taxa. Our results suggest that it is crucial to develop exact dynamic programming solutions for precise evolutionary inference, given that the accuracy of local search heuristics quickly deteriorates.

## Conclusion

Supertree approaches, and in particular median tree approaches, proved to be valuable tools for inference of phylogenetic species trees. Unfortunately, to the best of our knowledge, there is no median tree problem of interest that was shown to be efficiently solvable (i.e., in polynomial time). Therefore, in practice, median tree problems are typically addressed by using local search heuristics. Despite the vast popularity and often appealing scalability of such heuristics, they have several significant drawbacks. The most concerning drawback is that there is typically no guarantee on the quality of supertrees found by such heuristics; that is, computed supertrees can be arbitrarily far from the truth.

To overcome the drawbacks of local search heuristics, the method REXACT that solves median tree reconciliation problems has been proposed previously. REXACT is guaranteed to find the best median trees under a tree reconciliation cost function of interest. To do that REXACT requires an input of *rooted* gene trees. In practice, however, gene trees are typically unrooted. This factor incurs an additional layer of complexity to solving tree reconciliation problems. That is, since the tree reconciliation costs are defined for rooted gene trees, solving tree reconciliation problems naïvely (using REXACT) would require a full enumeration of all rooting combinations of the input trees. Such an approach is infeasible both in theory and in practice – as our scalability study shows.

We propose an exact novel method, UREXACT, that is capable of computing exact median trees for collections of unrooted gene trees. The dynamic programming algorithm developed in this work is enabled by the crucial so-called *plateau properties* [38]. Despite the intricacy of the developed algorithm, the asymptotic runtime of UREXACT is the same as the asymptotic runtime of REXACT when input gene trees are complete. Perhaps, most surprisingly, this implies that the runtime of UREXACT grows *linearly* as the number of input trees grows.

Finally, note that the proposed dynamic programming formulation can be now applied to much larger datasets (i.e., with hundreds of taxa) using the *cluster-constrained approach*. This approach entails selecting a set of clusters that are likely to appear in the “true” species tree, and constraining the dynamic programming procedure to work only with the selected clusters. This approach proved to be highly efficient, for example, with the popular ASTRAL software [52, 53]. The implementation of this powerful idea is part of our future research efforts.

## Data Availability

The software package is available at https://bitbucket.org/pgor17/urexact.

## Abbreviations

D: Duplication
L: Loss
DL: Duplication and loss
DC: Deep coalescence
uMinST: Unrooted minimal species tree
uMinCC: Unrooted minimal cost computation
rMinST: Rooted minimal species tree
rMinCC: Rooted minimal cost computation

## Footnotes

1 By “complete” we imply that the trees have full taxon sets.
